# Evidence for Genetic Nurture Effects on Substance Use

**DOI:** 10.1101/2025.08.28.25334658

**Published:** 2025-08-29

**Authors:** Mannan Luo, Victória Trindade Pons, Nathan A. Gillespie, Hanna M. van Loo

**Affiliations:** 1 Department of Psychiatry, University Medical Center Groningen, University of Groningen, Groningen, the Netherlands; 2 Virginia Institute for Psychiatric and Behavioral Genetics, Virginia Commonwealth University, Richmond, VA, USA

## Abstract

Substance use runs in families. Beyond genetic transmission, parental genetics can indirectly influence offspring substance use through the rearing environment, known as “genetic nurture”. This study utilized transmitted and non-transmitted polygenic scores to investigate genetic nurture effects on tobacco, alcohol and cannabis use in up to 15,863 adults with at least one genotyped parent from the Lifelines cohort. Genetic nurture significantly influenced smoking quantity (cigarettes per day: *β*=0.03; pack-years: *β*=0.02), accounting for 18.8% and 28.6% of the corresponding effects of genetic transmission. However, it had minimal impact on tobacco or cannabis initiation, suggesting a stage-specific pattern. Maternal and paternal genetic nurture contributed equally to offspring smoking quantity, especially for pack-years. Mediation analyses revealed that both maternal and paternal smoking partially explained these effects, with higher mediation proportions observed for maternal smoking quantity. These findings highlight the importance of considering stage-specific and parent-specific effects when investigating genetic nurture in substance use.

## INTRODUCTION

Substance use, including tobacco, alcohol and cannabis use, runs in families^1,2^. Parental substance use is one of the most salient familial risks for offspring’s substance use behavior and disorders^3–5^. Understanding the mechanisms underlying parent-offspring similarities is important for addressing intergenerational transmission of substance use. These similarities are attributed to both *nature* (the genes passed from parents to offspring) and *nurture* (the family environment created by parents)^6–8^. However, disentangling these influences remains challenging, particularly due to unmeasured genetic confounding in risk factors traditionally considered “environmental”^9^. Many environmental risk factors, including parental substance use, are partially genetic^10^, further complicating our understanding of substance use transmission across generations.

Advances in molecular genetics have made it possible to distinguish between transmitted and non-transmitted parental genotypes^11,12^. Parental transmitted alleles influence offspring outcomes both directly through inheritance and indirectly through the environment, while non-transmitted alleles affect offspring only indirectly through the rearing environment – a process known as “genetic nurture”^11,12^. For example, parents with a higher genetic risk for substance use may be more likely to engage in substance use themselves, thereby increasing the likelihood of offspring substance use indirectly through the rearing environment (e.g., modeling behavior, and accessibility of substances)^2,13^. To distinguish genetic transmission from genetic nurture, researchers calculate polygenic scores for transmitted (PGS_T_) and non-transmitted alleles (PGS_NT_), each score representing genetic predisposition for a given trait^11,12^. By examining PGS_T_ and PGS_NT_, we can determine how parental genetics shape both inherited risk and the rearing environments that predispose offspring to substance use.

While robust genetic nurture effects have been demonstrated for educational outcomes^14^, research on its role in substance use remains scarce. This study aims to disentangle genetic transmission and genetic nurture on substance use in adulthood. Specifically, we address three key questions: i) Do genetic nurture effects contribute to offspring substance use in adulthood? ii) Do these effects differ between mothers and fathers (i.e., parent-of-origin effects)? iii) To what extent are these effects mediated by familial environmental factors, such as parental substance use?

First, we investigated whether parental PGS_T_ and PGS_NT_ (aggregated across both parents) predict offspring tobacco, alcohol, and cannabis use in adulthood. Previous studies have identified genetic nurture effects, primarily in adolescence and young adulthood^15^ or in high-risk samples^16^ with a narrow focus of substance types (e.g., problematic alcohol use). However, it remains unclear whether these effects i) persist into adulthood, even after offspring leave the family household^17^; and ii) generalize to the broader population and across different substances. To address these gaps, we examined genetic nurture on a set of substance use measures in adult offspring from Lifelines, a general population cohort in the Netherlands. We hypothesized that PGS_NT_ would be associated with offspring substance use outcomes, providing evidence of genetic nurture, while PGS_T_ would explain a larger proportion of the variance.

Additionally, prior research has highlighted that relying solely on complete trio data (both parents and offspring genotyped) may introduce selection bias and limit generalizability^18^. To mitigate this, we utilized a validated haplotype-based approach^19^ to construct PGS_T_ and PGS_NT_ for smoking initiation (SmkInit), cigarettes per day (CigDay), alcoholic drinks per week (DrnkWk), and lifetime cannabis use (CanU). This method enables the inclusion of both parent-offspring pairs (one parent and their offspring are genotyped) and complete trios, increasing sample size and enhancing generalizability.

Second, we examined parent-of-origin effects with separate maternal and paternal PGS_T_ and PGS_NT_. Although both parents contribute to genetic nurture, their effects may differ in magnitude. Prior research suggests that paternal and maternal genetics similarly influence offspring educational attainment^11,14,19^, but mothers have stronger genetic nurture effects than fathers on health-related traits^11,20^ and attention problems^21^. However, no study has investigated parent-of-origin effects on substance use. This study directly addresses this gap by partitioning maternal and paternal genetic nurture effects, providing a more nuanced understanding of the roles of mothers and fathers in the intergenerational transmission of substance use. Given the absence of prior research in this area, our analysis was exploratory and did not include a specific hypothesis.

Third, we explored whether and to what extent parental substance use represents a mediating pathway through which genetic nurture contributes to offspring outcomes. While identifying genetic nurture reveals that parental genetics influences offspring outcomes through environmental pathways, it does not specify which environmental factors are involved. Mediation analysis helps to clarify these pathways by examining how parental genetic liability influences offspring outcomes through specific environmental factors. Parental substance use is a particularly promising mediator due to its role in offspring substance use and its partially genetic basis^3,6^. Previous studies have identified parental substance use as a mediator of genetic nurture in adolescents and young adults^15^ or high-risk families^16^. This study extends prior work by: i) distinguishing maternal and paternal mediating pathways, and ii) explicitly modeling parallel mediation pathways for both transmitted and non-transmitted genetics. We hypothesized that these genetic effects would be partially mediated by parental substance use.

## RESULTS

### Population characteristics

We utilized data from a total of 19,233 genotyped adult offspring with at least one parent genotyped from the Lifelines cohort, comprising 15,966 parent-offspring pairs and 3,267 trios. Of these, up to 15,863 participants (mean age=31.66 years; 61.9% female), consisting of 13,411 pairs and 2,452 trios, completed assessments for tobacco and alcohol use at baseline (2006–2013) and cannabis use during the second wave (2014–2017). Descriptive statistics for these participants are presented in Table 1. Notably, offspring with both parents genotyped (trios) reported significantly lower prevalence of smoking initiation, fewer cigarettes per day and pack-years, compared to those with only one genotyped parent (pairs). Correlations among PGSs and offspring outcomes are provided in Supplemental Table S1.

### Genetic nurture effects on substance use

We combined maternal and paternal PGS_T_ and PGS_NT_ for statistical power (see Supplemental Information). Linear mixed regression models were used to examine overall effects of PGS_T_ and PGS_NT_ (Table 2). Following Kong et al.^11^, we estimated the effect of direct genetic transmission (β_DGT_) by subtracting the effect of PGS_NT_ from that of PGS_T_ (i.e., *β*_*T*_ − *β*_*NT*_). This approach isolates “true” genetic transmission by removing the influence of genetic nurture, given that PGS_T_ may influence offspring directly through genetic inheritance and indirectly via genetic nurture.

As expected, each PGS_T_ was significantly associated with its corresponding substance use outcomes, with odds ratios between 1.3 and 1.9 for binary outcomes and standardized coefficients ranging from 0.09 to 0.19 for continuous outcomes. For genetic transmission effects, β_DGT_ was 0.16 for cigarettes per day, 0.07 for pack-years, and 0.10 for daily alcohol intake. For genetic nurture effects, parental PGS_NT_CigDay_ showed relatively smaller but significant associations with offspring cigarettes per day (*β*_*NT*_ = 0.03) and pack-years (*β*_*NT*_ = 0.02) after false discovery rate (FDR) correction. These genetic nurture effects equaled approximately 18.8% and 28.6% of β_DGT_ for cigarettes per day and pack-years, respectively. While parental PGS_NT_DrnkWk_ was significantly associated with offspring daily alcohol intake, this effect did not survive FDR correction. No associations found between parental PGS_NT_ and smoking initiation or lifetime cannabis use.

Overall, these findings indicate modest genetic nurture effects on smoking quantity. In contrast, initial tobacco and cannabis use, and daily alcohol intake were predominantly influenced by genetic transmission, with minimal contributions from genetic nurture.

### Parent-of-origin effects

Given the significant genetic nurture effects observed for smoking quantity, we further examined parent-specific effects on cigarettes per day and pack-years (Table 3). Structural equation modelling (SEM) was used to simultaneously estimate maternal and paternal PGS_T_ and PGS_NT_ effects, while accounting for potential genetic assortative mating on tobacco use^22^. Full-information maximum-likelihood estimation (FIML)^23^ was applied to handle missing data, enabling the inclusion of all 19,233 offspring from the full genotyped family sample.

Both maternal and paternal PGS_T_CigDay_ significantly predicted offspring smoking quantity, with paternal PGS_T___CigDay_ showing slightly greater effects. Wald tests indicated that the magnitude of PGS_T_CigDay_ did not differ significantly between mothers and fathers (*Δχ*^*2*^ = 3.35, *p* = .07 for cigarettes per day, *Δχ*^*2*^ = 2.49, *p* = .12 for pack-years).

For genetic nurture effects, both maternal and paternal PGS_NT_CigDay_ were significantly associated with offspring pack-years, with similar effect sizes (maternal *β* = 0.027, 95% CI [0.001, 0.052], paternal *β* = 0.034, 95% CI [0.001, 0.065]). Wald test confirmed no significant difference between maternal and paternal effects (*Δχ*^*2*^ = 0.01, *p* = 0.91). For cigarettes per day, maternal and paternal PGS_NT_CigDay_ were also nearly identical in magnitude, but the association was significant only for mothers (maternal 95% CI [0.008, 0.069], paternal 95% CI [−0.001, 0.071]). No significant associations were found between maternal and paternal PGSs (Supplemental Table S2), indicating little evidence of genetic assortative mating for smoking quantity. This suggests that estimates of genetic nurture effects were unlikely to be inflated by genetic similarity between parents.

### Mediation by parental tobacco use

To explore the extent to which parental tobacco use mediated the genetic nurture effects identified above, we conducted mediation analyses separately for mothers and fathers (Supplemental Information).

**Figure 1.**
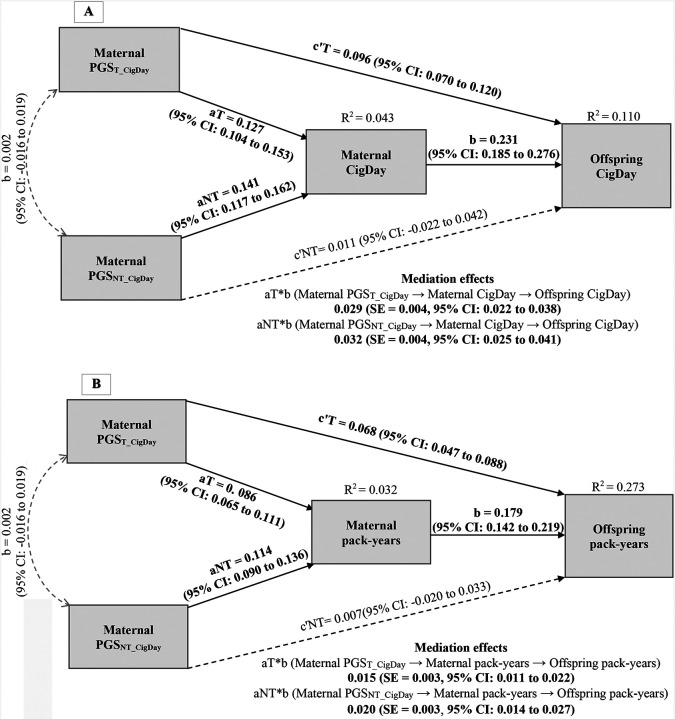

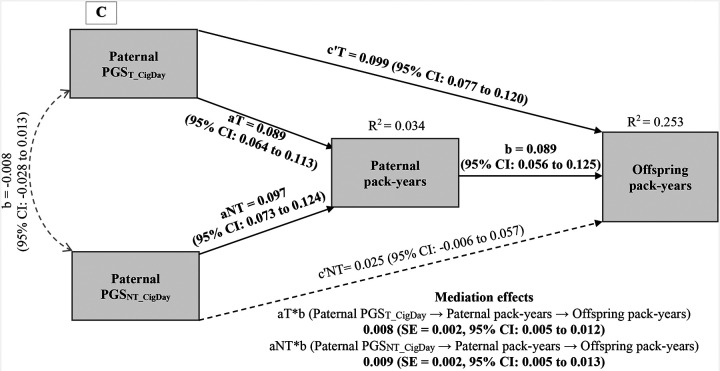
Mediation analysis using structural equation modeling: maternal/paternal smoking quantity as a mediator of the associations between transmitted (PGS_T_) and non-transmitted (PGS_NT_) polygenic scores and offspring smoking outcomes. Note. In all panels, aT path is PGS_T_ on maternal/paternal smoking quantity; aNT path is PGS_NT_ on maternal/paternal smoking quantity; b path is maternal/paternal smoking quantity on offspring smoking outcomes; c’T path is the direct effect of PGS_T_ on offspring smoking outcomes, while accounting for the mediator; and c’NT path is the direct effect of PGS_NT_ on offspring smoking outcomes, while accounting for the mediator. Mediation effects are presented below each mediation model for aT*b and aNT*b. Sex and age were included as covariates in all models. Solid line and bold indicate a significant pathway, and dashed line represent a non-significant (*p* > .05) pathway. **A. Maternal cigarettes per day** (CigDay). Maternal CigDay mediated the influence of both PGS_T_ (*β*_*total*_ = 0.125, SE = 0.012, 95% CI: 0.102 to 0.148) and PGS_NT_ (*β*_*total*_ = 0.043, SE = 0.016, 95% CI: 0.011 to 0.073) on offspring CigDay. **B. Maternal pack-years**. Maternal pack-years mediated the influence of both PGS_T_ (*β*_*total*_ = 0.084, SE = 0.011, 95% CI: 0.062 to 0.103) and PGS_NT_ (*β*_*total*_ = 0.027, SE = 0.013, 95% CI: 0.000 to 0.052) on offspring pack-years. **C. Paternal pack-years**. Paternal pack-years mediated the influence of both PGS_T_ (*β*_*total*_ = 0.107, SE = 0.011, 95% CI: 0.085 to 0.128) and PGS_NT_ (*β*_*total*_ = 0.034, SE = 0.016, 95% CI: 0.003 to 0.065) on offspring pack-years.

As shown in Figure 1(A, B), maternal smoking quantity significantly mediated the effects of both PGS_T_CigDay_ and PGS_NT_CigDay_ on offspring smoking outcomes, with mediated proportions of 23.2% (cigarettes per day: *β*_*mediation*_ = 0.029, *β*_*total*_ = 0.125) and 17.86% (pack-years: *β*_*mediation*_ = 0.015, *β*_*total*_ = 0.084) for PGS_T_, and 74.42% (cigarettes per day: *β*_*mediation*_ = 0.032, *β*_*total*_ = 0.043) and 74.07% (pack-years: *β*_*mediation*_ = 0.020, *β*_*total*_ = 0.027) for PGS_NT_. In contrast, paternal pack-years had a lower mediated proportion (Figure 1C), mediating 7.48% of PGS_T_CigDay_ (*β*_*mediation*_ = 0.008, SE = 0.002; *β*_*total*_ = 0.107) and 26.47% of PGS_NT_CigDay_ (*β*_*mediation*_ = 0.009; *β*_*total*_ = 0.034) on offspring pack-years. These findings suggest that parental smoking, especially maternal smoking, partially mediates genetic nurture effects on offspring smoking behaviors.

## DISSCUSION

This study aimed to disentangle genetic nurture and genetic transmission effects on substance use during adulthood, utilizing transmitted and non-transmitted polygenic scores. We examined these effects on various substance use traits, including across smoking initiation, smoking quantity (cigarettes per day, pack-years), daily alcohol intake, and lifetime cannabis use. Additionally, we investigated parent-of-origin effects and the mediating role of parental substance use to provide a nuanced understanding of intergenerational transmission underlying substance use. We highlight three key findings. First, genetic nurture significantly influenced smoking quantity, but not smoking initiation, daily alcohol intake, or lifetime cannabis use. As expected, genetic transmission played a substantial role across all measured outcomes. Second, parent-of-origin analyses revealed that the magnitudes of genetic nurture effects on smoking quantity were nearly identical for mothers and fathers. Finally, parental smoking quantity partially mediated the effects of both transmitted and non-transmitted genetic risk on offspring smoking outcomes in adulthood, with higher mediation proportions for maternal smoking quantity.

This study extends previous research by exploring genetic nurture on multiple substance use traits during adulthood within a large population-based cohort. While Saunders et al.^15^ reported genetic nurture effects on cigarettes per day at age 24 but not at age 29, our results indicate these effects persist into late adulthood, even after offspring have likely left the parental home. This discrepancy likely reflects differences in study design (twin study versus population-based study), sample age ranges (ages 17–29 versus ages 18–67), and the temporary dynamics of genetic nurture. Additionally, our study found no significant genetic nurture on smoking initiation or lifetime cannabis use, suggesting that such effects might be minimal on the initiation of substance use in adults. Several explanations may underlie this finding. First, the dichotomous measure used for initial use may be underpowered to detect genetic nurture effects, despite the large sample size for smoking initiation (*N* = 15,853). Continuous and cumulative metrics, such as cigarettes per day and pack-years, potentially enhance sensitivity to capture the persistence of genetic nurture into adulthood. Second, genetic nurture on substance use initiation may be more pronounced during adolescence, a critical period for the onset of such behaviors^24^, but these effects likely diminish by adulthood. This aligns with studies showing genetic and environmental contributions change over time^25,26^ and across different stages of substance use^6,7,27,28^, such as initiation, quantity/persistence, and dependence. For instance, shared environmental factors have consistently been shown to explain substantial variance in smoking initiation during early adolescence but contribute minimally to young adulthood^26,29^. Conversely, genetic factors explain an increasing proportion of variance in substance use as individuals age^25,30^. As genetic nurture partly mirrors these shared environment effects^31^, its effects may be limited for traits with low shared environmental estimates.

We found no evidence for genetic nurture on daily alcohol intake. This could be due to our short-term measurement of alcohol use (quantity and frequency over the past 30 days) which may not adequately capture long-term drinking patterns^32^. One study^16^ in a high-risk sample identified genetic nurture for alcohol use disorder, suggesting that genetic nurture may exist for pathological alcohol use but not for daily alcohol intake. Alcohol consumption, particularly measured in a general population cohort, may vary depending on social situations or specific life events (e.g., being a college student, celebrations or holidays). These contextual factors may contribute to why genetic nurture plays a less important role in daily alcohol intake.

Together, these findings provide valuable insights into the influence of genetic nurture on various types and stages of substance use. Genetic nurture appears to have a stronger influence on sustained or problematic substance use, such as smoking quantity and alcohol dependence involving regulation and reinforcement over time^33,34^. Conversely, its impact on initial experimentation, such as tobacco or cannabis initiation, may be minimal in adulthood. These observed patterns of genetic nurture, particularly their persistence into adulthood, align with developmental models^35^ emphasizing long-term parental influences on substance use behaviors. Future research should leverage longitudinal data to explore how genetic nurture effects unfold across the lifespan and across stages of substance use.

Another key contribution of this study is the investigation of parent-of-origin effects, revealing that maternal and paternal genetic nurture contribute equally to offspring smoking quantity. Although paternal genetic nurture was not statistically significant for cigarettes per day, likely due to reduced power from a smaller sample size, its overall effect was comparable to maternal genetic nurture. Our result aligns with previous findings of similar genetic nurture effects from mothers and fathers for other complex traits, including educational attainment^11,14^ and birth weight^36^. One plausible explanation is that both parents are equally important in shaping shared family environments and parenting practices, which in turn influence offspring smoking outcomes. Consistent with this, previous evidence suggests that substance use intervention efforts may benefit from considering both maternal and paternal influences^37,38^.

Interestingly, despite maternal and paternal genetic nurture effects on pack-years were nearly equal at the genetic level, our mediation analyses showed maternal smoking quantity constitutes a more substantial mediating pathway in both relative terms (mediating approximately 74% versus 26% of genetic nurture effects) and absolute magnitude (mediation effect of 0.020 versus 0.009). This suggests that the mechanisms through which genetic nurture acts on offspring may differ between parents. Our findings highlight the importance of dissecting parent-specific mediation pathways, as maternal and paternal genetic nurture may impact offspring behaviors through distinct mechanisms. Mothers may exert genetic nurture effects on offspring smoking primarily through accumulative smoking exposure (e.g., prenatal and postnatal), aligning with research linking such exposure to offspring substance use^3,39^. Fathers’ influence may operate through other pathways, such as paternal psychiatric disorders^40^ and father-child relationship^41^, beyond direct smoking exposure, which were not included in our mediation model. Future research should explore alternative mediation pathways to gain a more comprehensive understanding of intergenerational transmission of substance use. Such findings might inform interventions aimed at reducing substance use risks through targeted parental support strategies.

This study has several strengths, including a large, population-based cohort and the use of a unique dataset integrating genotypic data with various substance use measures from mothers, fathers, and offspring. Unlike much existing research that combines parental PGS, this study partitioned maternal and paternal genetic effects, allowing for the investigation of parent-of-origin genetic nurture. Additionally, by differentiating between transmitted and non-transmitted alleles, we were able to identify genetic nurture effects unconfounded by direct genetic transmission. However, several limitations remain. First, our sample included only participants of European descent, potentially limiting generalizability. Second, PGSs explain only a small proportion of genetic liability to substance use, and the modest genetic nurture effect sizes observed reflect this limitation. Third, paternal analyses may be underpowered due to limited availability of fathers with both genotypic and phenotypic data. Fourth, retrospective self-reports on substance use, such as pack-years, may be subject to recall bias or underreporting^42^. However, measures like cigarettes per day and pack-years have demonstrated strong reliability and validity in assessing lifetime smoking exposure^43^. Fifth, we were unable to examine genetic nurture effects across different age groups due to sample size constraints, despite evidence suggesting that such effects may vary with age^15,44–46^. Finally, our hypothetical mediation models were theory-driven but not exhaustive. Comparisons with saturated models (Supplemental Table S3), which perfectly fit the data by estimating all possible paths, showed better statistical fit for the saturated models across all analyses (*p* < .01). While this suggests potentially unmodeled paths, these comparisons should be interpreted in light of our large sample size, which increases sensitivity to detect even minor modelling misspecifications. Notably, the differences in sample-size-insensitive fit indices were minimal (ΔRMSEA = 0.02, ΔCFI = −0.01 to −0.004), suggesting our theoretically-grounded mediation models maintain acceptable fit while offering greater parsimony.

## CONCLUSIONS

To conclude, we provide evidence for genetic nurture on smoking behaviors in adulthood with a stage-specific pattern, while direct genetic transmission remains the primary driver of substance use. We found that maternal and paternal genetic nurture contribute equally to offspring smoking quantity, particularly pack-years. Moreover, parental substance use mediates genetic nurture effects, with maternal smoking quantity potentially playing a larger mediating role. Our study provides a nuanced understanding of genetic nurture effects on substance use, which might operate in stage-specific and parent-specific ways. These findings, particularly the persistence of genetic nurture effects into adulthood, highlight the importance of both maternal and paternal influences in research and interventions aimed at reducing the intergenerational transmission of substance use.

## METHODS

### Participants

Lifelines is a multi-disciplinary prospective population-based cohort study examining in a unique three-generation design the health and health-related behaviors of 167,729 persons living in the North of the Netherlands. It employs a broad range of investigative procedures in assessing the biomedical, socio-demographic, behavioral, physical and psychological factors which contribute to the health and disease of the general population, with a special focus on multi-morbidity and complex genetics^47,48^. Data collection was accomplished at three general assessments, along with additional assessments. The baseline assessment took place between 2007 and 2013, followed by a second wave between 2014 and 2017, and a third wave between 2019 and 2023. The design and sample characteristics of Lifelines have been described in detail elsewhere^47,48^.

### Measurements

#### Substance use

Substance use was measured by self-report questionnaires at baseline for adult offspring and their parents, except for cannabis use which was measured at the second wave. Smoking initiation was defined as having smoked for one year or longer with the question “Have you ever smoked for a full year in your lifetime?”. Participants who smoked for less than a year were not considered as a smoker. In participants who smoked for a year or more, smoking quantity was assessed in cigarettes per day and pack years. Cigarettes per day was defined as the lifetime average number of cigarettes smoked per day, either as a current or former smoker. Pack-years was calculated by multiplying the average amount smoked per day (different types of tobacco, including cigarettes, cigarillos, cigars and pipes) by the number of years the person smoked in their lifetime (1 pack-year equals 20 cigarettes per day for one year). Pack-years is a cumulative indicator of lifetime smoking behavior (not only singular tobacco use variables like cigarettes per day, but also other tobacco types).

Alcohol use was assessed with a food frequency questionnaire developed by Wageningen University^49^. Two questions referred to the frequency and quantity of alcohol consumed in the past month: “How often did you drink alcoholic drinks in the past month?” (ranging from ‘not this month’ to ‘6–7 days per week’), and “On days that you drank alcohol, how many glasses did you drink on average?” (from ‘1’ to ‘12 or more’). These questions were split up for different alcoholic groups (beer, alcohol-free beer, red wine/rose, white wine, sherry, distilled wine, other alcoholic beverages). Based on these questions, an average daily alcohol consumption in grams per day was calculated^50^. This composite index of daily alcohol intake provides a more comprehensive measure of overall alcohol use than a single measure of frequency (e.g., number of drinking days per month) or quantity (e.g., glasses per day).

Lifetime cannabis use was defined using two questions: i) “Have you ever used drugs?”, and if yes, ii) “Have you ever used cannabis, such as weed, marijuana, hashish?”. The answer categories were recoded to ever (1) versus never (0) used cannabis.

#### Genotyping and imputation

A total of 79,988 participants were genotyped across three batches in Lifelines. Quality control (QC) of markers and samples was performed separately per batch. Detailed pre-imputation QC criteria is described in Supplemental Information.

In brief, markers that were duplicated and monomorphic, markers with a low call rate or low minor allele frequency, and markers that deviated significantly from Hardy-Weinberg equilibrium were removed. Post QC data from each array was imputed through the Sanger Imputation Service with the Haplotype Reference Consortium v1 panel. We selected overlapping imputed markers with quality scores ≥0.8 across arrays to create a common set of markers for all genotyped parents and offspring in any arrays. Samples with a low call rate, heterozygosity outliers or mix-ups on sex and familial relationship were filtered out. Samples were restricted to participants of European ancestry, determined through principal component analysis with the 1000 Genomes reference, to control for population stratification.

#### Non-transmitted alleles inference

We applied our newly developed haplotype-based approach to differentiate transmitted and non-transmitted alleles in genotyped parent-offspring pairs and trios^19^. By including parent-offspring pairs, rather than restricting the analysis to trios, this approach reduces sample attrition and improves statistical power. The development and validation of this method have been described in detail elsewhere^19^. Briefly, we used SHAPEIT5 to estimate haplotypes including pedigree information^51^. Offspring haplotypes were then compared to parental haplotypes using tiles of 150 adjacent markers on each chromosome. The best match between the parent and offspring tiles, taking recombination spots into account, was used to determine which parental tiles were transmitted to the offspring. The remaining non-transmitted alleles were recorded in a separate dataset, and for parent-offspring pairs, the non-transmitted alleles of the parent who was not genotyped were set as missing. This method was validated by comparison with standard software in parent-offspring trios and found a concordance rate for the non-transmitted alleles of 99.8%. Furthermore, the identification of non-transmitted alleles was confirmed to be unaffected by missing parental data through simulations of pairs from trios.

#### Polygenic scores

We calculated PGS_T_ and PGS_NT_ based on summary statistics from previous genome-wide association studies (GWAS) for SmkInit^52^, CigDay^52^, DrnkWk^52^, and CanU^53^ (Supplementary Table S4). These GWAS were chosen because they were based on the largest sample sizes currently available for each corresponding phenotype in Lifelines.

To increase the variance explained by each PGS, SNP effects were re-weighted using the ‘auto’ setting from LDpred2^54^, a Bayesian method that adjusts the effect estimates from GWAS summary statistics by incorporating trait-specific genetic architecture (e.g., SNP-based heritability and polygenicity measured as the fraction of causal variants) and linkage disequilibrium (LD) data from UK Biobank reference panel for European ancestry^55^. For each offspring, PGSs were created based on transmitted and non-transmitted datasets. To estimate overall genetic nurture and genetic transmission effects, parental PGS_T_ and PGS_NT_ were defined as the sum of the PGS based on paternal and maternal transmitted and non-transmitted haplotypes, respectively. The value of the missing PGS_NT_ in parent-offspring pairs was imputed with the average PGS_NT_ of the observed parents (Supplementary Information). To estimate parent-of-origin effects, we separated four maternal and paternal PGS_T_ and PGS_NT_ respectively. To control for population structure and batch effects across arrays, we standardized PGS residuals within each array after regressing out the first ten genetic principal components.

### Statistical analysis

All analyses were conducted in R^56^. We applied a stepwise approach in which genetic nurture effects had to be statistically significant to continue to the next analysis.

#### Genetic nurture and genetic transmission on substance use

First, we used mixed-effects regression models to examine associations between parental PGS_T_ and PGS_NT_ with offspring substance use outcomes, including smoking initiation, smoking quantity (cigarettes per day, pack-years), daily alcohol intake, and lifetime cannabis use. Continuous outcomes were analyzed using mixed-effects linear regression with the ‘*lme4*’ package^57^, while dichotomous outcomes were analyzed using mixed-effects logistic regression with the ‘*GLMMadaptive*’ package^58^. Each model was specified as follows: *Y*_*i*_ = *Intercept*_*Y*_ + *βPGS*_*T*_ + *βPGS*_*NT*_ + *sex* + *age* + 1|*FamilyID* + *e*_*i*_. Family ID was included as a random effect (intercept) to account for the relatedness among siblings, along with sex and age as covariates. False discovery rate (FDR) corrections^59^ (5 tests, α = 0.05) were applied to control for multiple testing across the two mixed-effects logistic regression models (smoking initiation, lifetime cannabis use) and three mixed-effects linear regression models (cigarettes per day, pack-years, and daily alcohol intake).

#### Parent-of-origin effects

If genetic nurture effects remained significant after FDR, we examined parent-of-origin effects on offspring’s substance use outcomes using structural equation modeling (SEM) in ‘*Lavaan’* package^60^. SEM enabled us to handle missing data with Full Information Maximum Likelihood(FIML), which utilizes all available data to estimate parameters and standard errors without imputing missing values^23^. For each SEM model, *Y*_*i*_ = *Interceot*_*Y*_ + *βPGS*_*T*_*mother*_ + *βPGS*_*NT*_*mother*_ +*βPGS*_*T*_*father*_ +*βPGS*_*NT*_*father*_ + *sex* + *age* + *e*_*i*_, all paths and covariances will be freely estimated. To account for non-normality and non-independence of observation^61^, we generated standard errors and 95% confidence intervals (CIs) using bootstrapping with 1,000 replications. To assess whether the maternal and paternal effects differed significantly, we compared the equality of standardized regression coefficients using a Wald test^62^, via *‘lavTestWald’* function in Lavaan.

#### Mediation pathways via parental substance use

Finally, we conducted mediation analysis using SEM (*Lavaan* package) to assess the extent to which parental substance use mediates the associations of PGS_T_ and PGS_NT_ with offspring substance use outcomes. FIML was used to handle missing data and reduce the likelihood of biased parameter estimates. Standardized mediation effects were estimated via bootstrapping with 1,000 replications, with statistical significance determined by 95% bootstrap CIs excluding zero. Additionally, we calculated the proportion mediated, which represents the percentage of the total effect on the outcome explained by mediation.

To assess the appropriateness of mediation model, we compared our hypothetical mediation models with saturated models, which perfectly fit the data by estimating all variances, covariances, and means of the observed variables (Supplementary Information).

## Supplementary Material

Supplement 1

## Figures and Tables

**Table 1.** Descriptive characteristics of Lifelines participants, including the subsamples of adult offspring from genotyped family parent-offspring pairs and trios

	All		Pairs		Trios		*p* *
	*N*	M±SD or n (%)	*N*	M±SD or n (%)	*N*	M±SD or n (%)	
Age (years) at assessment	15871	31.66 ± 8.61	13417	30.59 ± 8.02	2454	31.86 ± 8.70	
Sex, female	15871	9827 (61.9)	13417	8376 (62.4)	2454	1451 (59.1)	
**Tobacco use**							
Smoking initiation, yes	15853	6560 (41.4)	13403	5620 (41.9)	2450	940 (38.4)	<.01
Cigarette per day	5972	10.24 ± 6.00	5134	10.34 ± 6.06	838	9.59 ± 5.57	<.01
Pack-years	6276	7.03 ± 6.73	5382	7.19 ± 6.85	894	6.04 ± 5.88	<.001
**Alcohol use**							
Daily alcohol intake (grams/day)	15863	6.74 ± 8.46	13411	6.73 ± 8.47	2452	6.81 ± 8.41	0.68
**Cannabis use**							
Lifetime cannabis use, yes	9097	2066 (22.7)	7608	1710 (22.5)	1489	359 (24.1)	0.18

Note: *N*= Sample size, M= Mean, SD = Standard deviation.

* Chi-square test for binary outcomes and t-test for continuous outcomes when comparing the pairs versus trios on substance use. Pack-years of smoking were calculated by multiplying the amount smoked per day (of different types of tobacco products, including cigarettes/roll-ups, cigarillos, cigars. grams of pipe tobacco) by the number of years the person has smoked.

**Table 2.** Regression coefficients of parental transmitted, non-transmitted polygenic scores, and direct genetic effects on offspring substance use in adulthood

		PGS_T_				PGS_NT_				DGT
	*N*	*β*_*T*_ (SE)/OR	95% CI	*p*	*p*_*FDR*_	*β*_*NT*_ (SE)/OR	95% CI	*p*	*p*_*FDR*_	*β*_*T*_ − *P*_*NT*_
**PGS**_**SmkInit**_										
Smoking initiation	15853	**1.853** ^a^	1.767, 1.944	<.0001	<.0001	.989^a^	.950, 1.031	.612	.612	–
**PGS**_**CigDay**_										
Cigarettes per day	5972	**.185 (.012)**	.162, .208	<.0001	<.0001	**.033 (.012)**	.011, .057	.004	.020	.16
Pack-years	6276	**.084 (.006)**	.071, .098	<.0001	<.0001	**.017 (.007)**	.004, .031	.014	.035	.07
**PGS**_**DrnkWk**_										
Daily alcohol intake	15863	**.120 (.007)**	.106, .134	<.0001	<.0001	.015 (.007)	.001, .031	.034	.057	.10
**PGS**_**CanU**_										
Lifetime cannabis use	9097	**1.274** ^a^	1.188, 1.365	<.0001	<.0001	1.033^a^	.967, 1.104	.328	.410	–

Note. Mixed-effects regression models include up to 15,863 adult offspring with at least one parent genotyped and available data on substance use outcomes. Age and sex were included as covariates, and sibling effect was controlled for by including family ID as a random effect into models. *β*_*T*_ *and β*_*NT*_: standardized coefficients of the polygenic scores calculated for the transmitted and non-transmitted alleles, respectively, when they are analyzed jointly. DGT= *β*_*T*_ − *β*_*NT*:_ estimated effect of genetic transmission of the polygenic score. SE=standard error, OR = odds ratio, 95% CI = 95% confidence interval*, p* = unadjusted p value, *p*_*FDR*_ = *p* value after FDR correction, SmkInit = smoking initiation, CigDay = cigarettes per day, DrnkWk = drink per week, CanU = lifetime cannabis use.

a the estimated odds ratio for smoking initiation and lifetime cannabis use from logistic regression models.

**Table 3.** Parent-of-origin effects on offspring smoking quantity: comparison of transmitted and non-transmitted polygenic scores for cigarettes per day split by paternal and maternal haplotypes

	Maternal PGS_T_CigDay_			Maternal PGS_NT_CigDay_			Paternal PGS_T_CigDay_			Paternal PGS_NT_CigDay_		
	*β* (SE)	95% CI	*p*	*β* (SE)	95% CI	*p*	*β* (SE)	95% CI	*p*	*β* (SE)	95% CI	*p*
Cigarettes per day	**.124 (.012)**	.100, .145	<.0001	**.039 (.015)**	.010, .069	.008	**.155 (.012)**	.131, .179	<.0001	.036 (.020)	.000, .076	.066
Pack-years	**.082 (.011)**	.062, .104	<.0001	**.027 (.013)**	.001, .052	.043	**.107 (.011)**	.085, .127	<.0001	**.034 (.016)**	.001, .065	.036

Note. Structural equation modeling was used to assess the effects of maternal and paternal transmitted (PGS_T_) and non-transmitted (PGS_NT_) polygenic scores on offspring smoking outcomes. As the analyses used a full information maximum likelihood (FIML) approach to handle missing data, retaining all available data from the full genotyped family sample (*N*=19,233), there was no list-wise *N* for the sample. Standardized coefficients (*β*), bootstrapped standard errors (SE), bootstrapped 95% confidence intervals (CIs) and *p* value were reported. FDR correction was not applied to the parent-of-origin analyses given the high correlation between outcome measures (cigarettes per day and pack-years), which would make such correction overly conservative for non-independent tests.

## Data Availability

Data may be obtained from a third party and are not publicly available. Researchers can apply to use the Lifelines data used in this study. More information about how to request Lifelines data and the conditions of use can be found on their website (https://www.lifelines.nl/researcher/how-to-apply).
